# The efficacy and safety of dexmedetomidine in preventing emergence delirium in paediatric patients following ophthalmic surgery: a systematic review and meta-analysis of randomised controlled trials

**DOI:** 10.1186/s44158-022-00079-y

**Published:** 2022-12-12

**Authors:** Hind M. Alassaf, Amal M. Sobahi, Nasser S. Alshahrani

**Affiliations:** 1 Queen’s University, Belfast, North Ireland UK; 2 Anesthesia Technology Department, College of Applied Medical Sciences, Imam Abdulrahman Bin Faisal University, Jubail, Saudi Arabia; 3 College of Applied Medical Sciences, King Khalid University, Abha, Saudi Arabia

**Keywords:** Dexmedetomidine, Emergence delirium, Emergence agitation, Paediatric, Strabismus surgeries, Cataract surgeries, Ophthalmic surgeries

## Abstract

**Background:**

The leading cause of emergence delirium (ED) in children postoperatively is the exposure to inhalational anaesthetics. ED can occur immediately after waking from anaesthesia, making patients generally uncooperative and agitated. Dexmedetomidine has sedative and analgesic effects and helps to reduce agitation and delirium and improve hemodynamic stability and the recovery of respiratory function; in addition to decreasing pain intensity, it is also well known for helping reduce nausea and vomiting.

**Objectives:**

This updated systematic review meta-analysis investigate and summarise currently available evidence on the use of dexmedetomidine to prevent ED, reduce postoperative nausea and vomiting (PONV) and decrease the need for rescue analgesia in paediatric patients undergoing ophthalmic surgery.

**Methods:**

The medical databases EMBASE, PubMed and Cochrane Library were searched for randomised controlled trials published between January 2020 and August 2022 that used Dexmedetomidine in paediatric patients undergoing ophthalmic surgery. The protocol was prospectively registered with PROSPERO (CRD42022343622). The review was accomplished according to the ‘Preferred Reporting Items for Systematic Reviews and Meta-Analyses’, and the meta-analysis was conducted by using RevMan5.4. These studies examine the efficacy of dexmedetomidine in preventing ED in children undergo ophthalmic surgery. The Cochrane ROB-1 was used to assess risk of bias (ROB).

**Results:**

Eight studies comprised of 629 participants, of which 315 received dexmedetomidine and 314 placebos were examined. PAED score identified ED following surgery. A review and meta-analysis indicated that dexmedetomidine reduces ED incidence (RR = 0.39; 95% CI 0.25–0.62). Similarly, it reduces the use of rescue analgesia (RR = 0.38; 95% CI 0.25–0.57). However, dexmedetomidine did not help prevent PONV since no difference was found between groups (RR = 0.33; 95% CI 0.21–0.54).

**Conclusion:**

This review showed that dexmedetomidine helped to reduce ED incidence in paediatric patients after ophthalmic surgery and reduced the need for rescue analgesia compared to placebo or other medications.

## Introduction

Emergence delirium (ED) is a combination of perceptual disturbance and psychomotor agitation, commonly occurring in preschool-aged children during early recovery after anaesthesia [1]. ED was first described in paediatric patients in the early 1960s [2]. However, ED can occur immediately after waking from anaesthesia, making patients generally uncooperative, irritable, incoherent, inconsolable and uncompromising with moaning and thrashing or kicking [3]. Moreover, parents are frequently anxious about anaesthetic and surgical complications, making ED distressing for them [4]. ED episodes are generally short-lived but can increase the risk of self-injury and delay discharge, requiring more nursing care and increasing medical care expenses [1, 2]. The leading causes of ED are varied, including the choice of inhalational anaesthetics agents. Sevoflurane and desflurane tend to increase ED incidence compared to halothane or isoflurane. Moreover, ear, nose and throat surgeries have been identified as a risk factor for ED [4]. However, a recent meta-analysis by Dahmani et al. found that using an α2-adrenergic receptor agonist such as dexmedetomidine had a prophylactic effect in EA prevention [5].

Dexmedetomidine has sedative and analgesic effects. It was approved by the US Food and Drug Administration in late 1999 and has since been used largely in the paediatric population due to its high selectivity for the α2-adrenergic receptor [6]. Dexmedetomidine help to reduce agitation and delirium and improve hemodynamic stability and the recovery of respiratory function [7]. Moreover, many recent studies have shown that dexmedetomidine can relieve postoperative pain. In addition to decreasing pain intensity, it is also well known for helping reduce nausea and vomiting [8–10].

This systematic review and meta-analysis investigate and summarise current available evidence on the use of dexmedetomidine to prevent ED, reduce postoperative nausea and vomiting (PONV) and decrease the need for rescue analgesia in paediatric patients undergoing ophthalmic surgery.

## Methodology

### Protocol and registration

This systematic review and meta-analysis were performed according to the Preferred Reporting Items for Systematic Reviews and Meta-Analyses (PRISMA) guidelines [11]. The protocol for this study was registered in PROSPERO (CRD42022339849).

### Inclusion and exclusion criteria

#### Study types

Only randomised controlled trials (RCTs) were included.

#### Participants types

Paediatric patients aged < 14 years scheduled for ophthalmic surgery.

#### Intervention types

Dexmedetomidine was used with no restrictions on the dose or route of administration compared to placebos or any other medication.

#### Exclusion

Excluded studies included all non-RCTs, those using dexmedetomidine in adults or for non-ophthalmic surgeries and those with only abstracts available, not written in English, or duplicated.

#### Search strategy

The studies were retrieved from the electronic medical PubMed, Cochrane and EMBASE databases for this review. The search was independently performed by all authors using the keywords ‘dexmedetomidine’, ‘emergence delirium’, ‘emergence agitation’, ‘paediatric’, ‘strabismus surgeries’, ‘cataract surgeries’ and ‘ophthalmic surgeries’ with the Boolean AND operator. The final search was performed on 20 July 2022 in all the databases*.* Only studies published between January 2020 and August 2022 passed the filtering process.

#### Study selection

The Endnote software was used to identify and remove duplicates, confirmed by manual screening. All authors screened the results independently to filter non-relevant articles based on their titles and abstracts. The remaining studies were then read in full to assess their eligibility. If the study had three arms and used a placebo in one comparator group, the placebo group was used as the comparator for dexmedetomidine. When two arms used dexmedetomidine with different doses, the arm with the dose closest to the other included studies was selected: dexmedetomidine at 1–2 mcg/kg intranasally or 0.3–1 mcg/kg intravenously.

#### Data collection processes

A data extraction form was used by all authors to independently extract data from the selected publications. Any disagreements were resolved through discussion to reach a consensus. The extracted data included information on the study (year of publication, authors, study design, country, setting and delirium assessment methods), participants (number, sex and age), intervention (dose, route and administration time) and controls (comparator types, doses and administration route).

#### Outcomes

The primary outcome was ED incidence. Secondary outcomes included rescue analgesia use and PONV.

#### Bias risk

The Cochrane Risk of Bias-1 (ROB-1) tool was used independently by all authors to assess bias risk. ROB-1 was used to assess potential bias sources in RCTs, including the selection, performance, detection, attribution and reporting bias. Their bias risk was then rated as high’, ‘low’ or ‘unclear’.

#### Statistical analysis

The data (the number of patients meeting each outcome) was extracted from the selected articles using a data extraction form and then meta-analysed using the Review Manager (RevMan) software. The primary outcome was ED incidence (%) diagnosed based on a score of >10 on the Paediatric Anaesthesia Emergence Delirium (PAED) scale. The secondary outcomes were rescue analgesia use (%) and PONV (%).

Dichotomous data were analysed using the Mantel–Haenszel test with random effects models to create forest plots and calculate an overall risk ratio (RR) with a 95% confidence interval (CI) for ED incidence, rescue analgesia and PONV. All *Z* test results with *p* < 0.05 were considered statistically significant. Heterogeneity was assessed with *I*^2^ tests, with values ≥75% considered highly heterogeneous, while values of 50–75% were considered moderately heterogeneous.

## Results

### Literature search and study evaluation

**Fig. 1:**
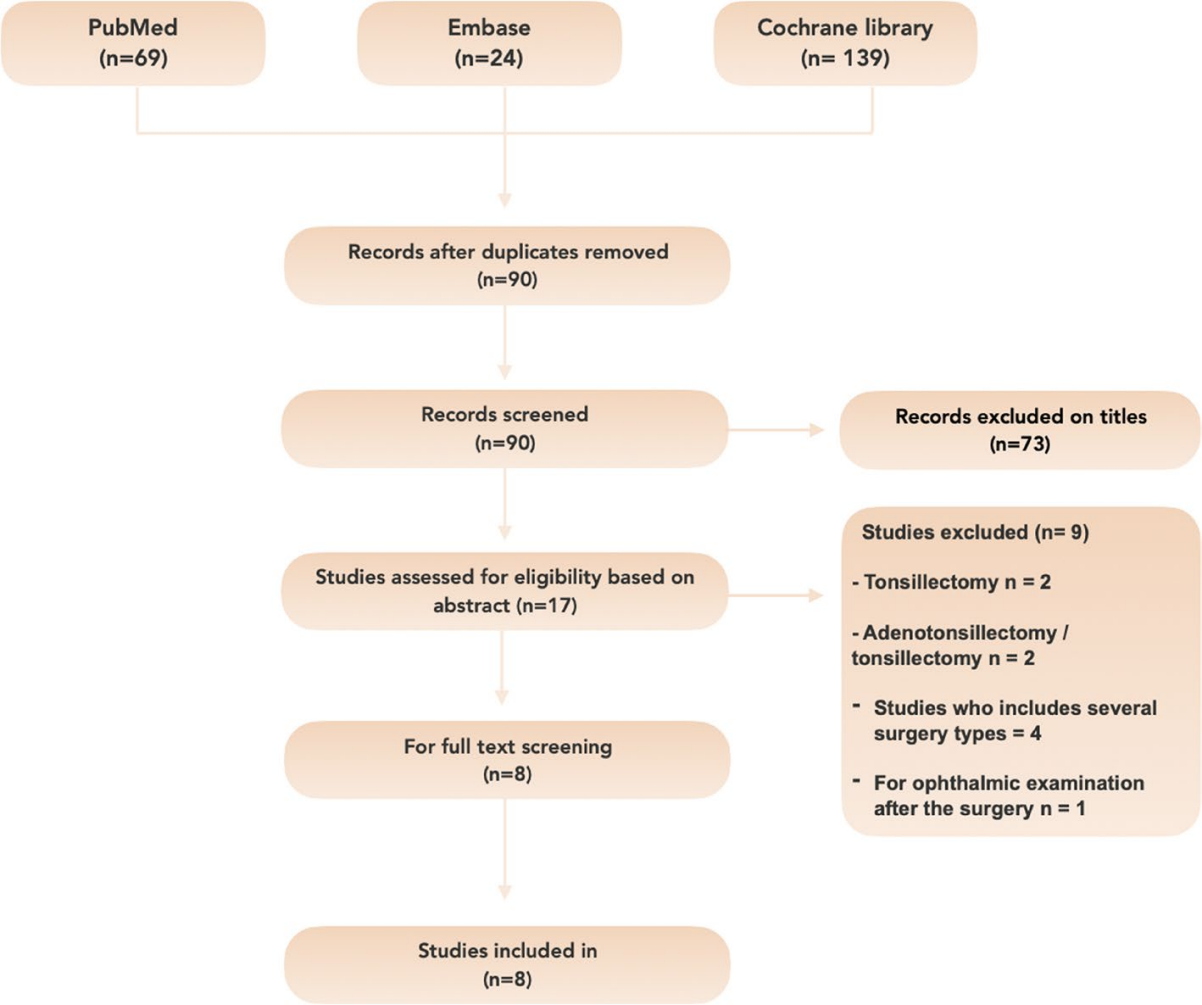
Modified PRISMA flowchart illustrating the systematic search and selection process

Ninety studies published between January 2020 and August 2022 were identified after removing duplicates using EndNote. After title screening, 73 studies were excluded, leaving 17 for abstract review. After abstract reading, nine studies were excluded, leaving 8 for full-text review. After full-text review, eight studies met the primary inclusion criteria and were found eligible for inclusion in this review (Fig. 1).

### Study characteristics

#### Included studies

Patient characteristics were thoroughly examined for all eight included studies [12–19], which were all prospective, double-blinded, parallel-group and single-centre RCTs. They were published in 2020 (*n*= 4), 2021 (*n*= 2) or 2022 (*n*= 2) and were all national studies conducted in China (*n*=3), India (*n*=2), Indonesia (*n*=1), Egypt (*n*=1) and Qatar (*n*=1). They included paediatric patients who underwent strabismus surgery (*n*=5), cataract surgery (*n*=1) or general ophthalmic surgery (*n*=2).

These eight studies comprised 629 participants, of which 315 received dexmedetomidine and 314 were controls. Participant ages were between 2 months and 14 years and were more often male (*n*=329; 52%) than female (*n*=300; 48%). In seven studies, dexmedetomidine was given preoperatively [12–17, 19], and in one study, it was given postoperatively [18]. Dexmedetomidine was administered intranasally in five studies [12–14, 17, 19] and intravenously in three studies [15, 16, 18].

General anaesthesia was started with 5–8% sevoflurane in seven studies [12–17, 19] and with 2 mg/kg propofol in one study [18]. Following the surgery, ED was assessed using the PAED scale and diagnosed if the patient had a score >10 [12, 14, 15, 17–19]. PAED score is a valid and reliable rating scale developed to standardise the ED evaluation to improve reporting and comparison of findings between studies [15, 16]. The PAED score has the additional benefit of accurately differentiating between postoperative pain and ED, which might present similarly [17]. However, paracetamol was administered postoperatively in five studies to ensure adequate analgesia and prevent the possible influence of pain on PAED scores in the dexmedetomidine and control groups [12, 15–17, 19].

**Fig. 2:**
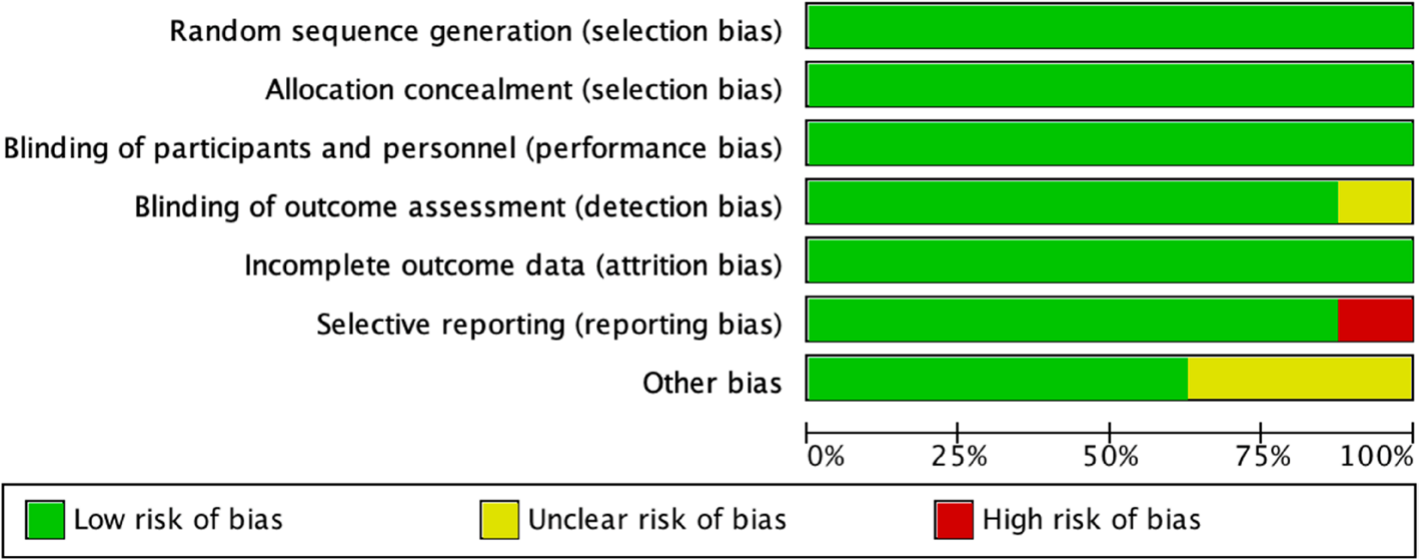
Bias risk assessment using RoB 1

**Fig. 3:**
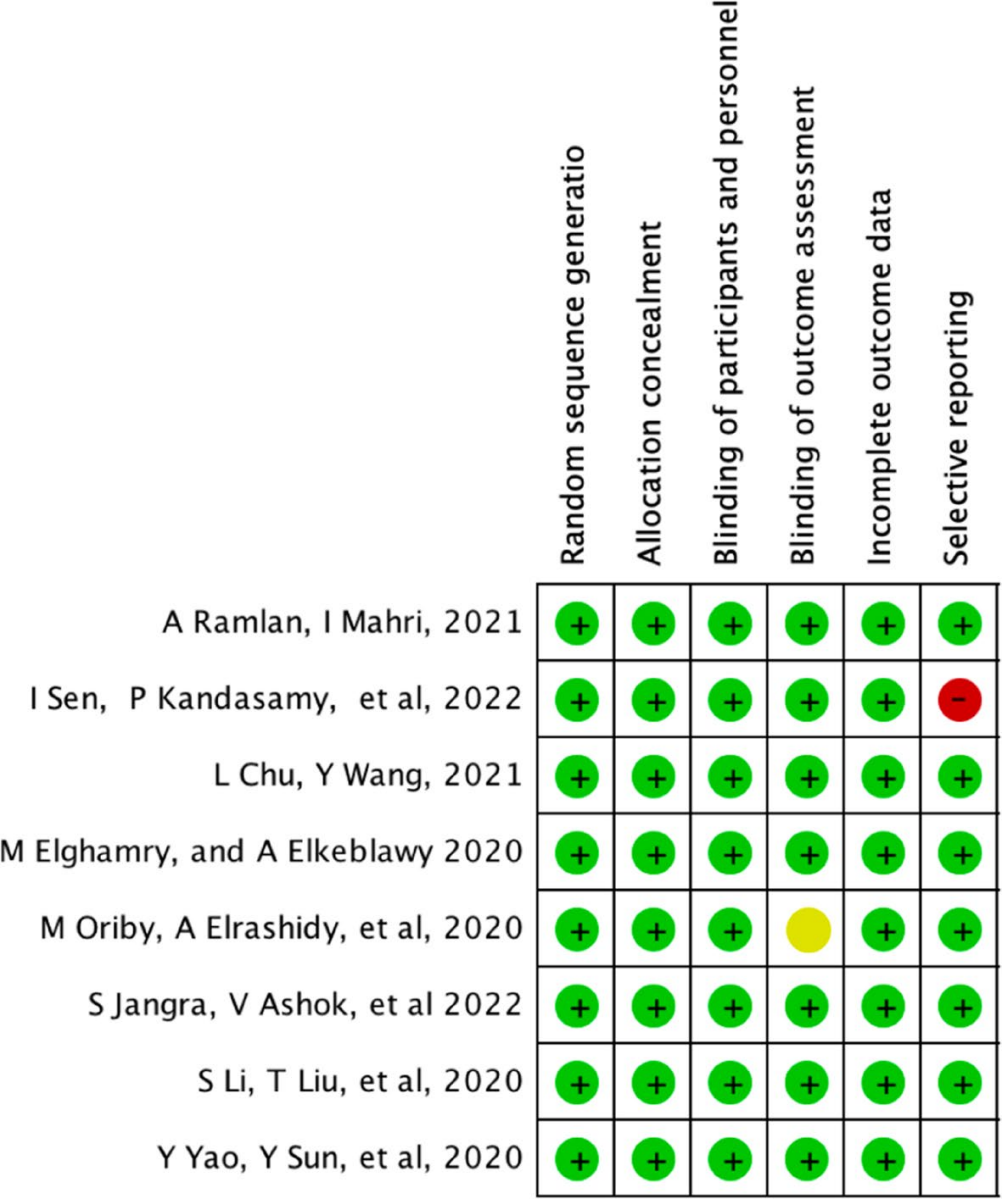
Bias risk summary using RoB 1

Postoperative pain were assessed using the faces, legs, activity, cry and consolability ability (FLACC) score in three studies [13, 16, 17]. If the score was >3, 0.5μg kg^-1^ fentanyl was administered as rescue analgesia [13, 17]. On the other hand, Yao et al. used a modified Children’s Hospital of Eastern Ontario Pain Scale to assess the postoperative pain level. If the score was >3, morphine was administered intravenously as rescue analgesia with dose of 25 ug kg^-1^ [19]. In addition, 0.1 mg kg^-1^ ondansetron was given intravenously as an antiemetic to all the patients in both groups postoperatively [12, 15, 17] (Tables 1 and 2; Figs. 2 and 3).

**Table 1:** Characteristics of the eight included studies

Author	Study design	Surgery type	Study country& centre	Intervention & comparators	Time of administration	Anesthesia induction	Doses		Sample size	
							D	C	C	D
Ramlan et al. [12]	**Prospective single-centre, double-blinded, parallel-group RCT**	**Ophthalmic**	**Indonesia (February to May 2019)**	**Intranasal dexmedetomidine vs midazolam**	**Preoperatively**	**6–8% sevoflurane**	**1 mcg kg**^**−1**^	**0.1 mg kg**^**−1**^	**32**	**32**
Sen et al. [13]	**Prospective single-centre, double-blinded, parallel-group RCT**	**Cataract**	**India**	**Intranasal midazolam + dexmedetomidine vs midazolam + normal saline**	**30–45 min before the surgery**	**8% sevoflurane**	**1 µg kg**^**−1**^ ** + 0.25 mg kg**^**−1**^** oral midazolam**	**0.5 mg kg**^**−1**^ ** + 0.025 mg kg**^**−1**^** saline**	**40**	**40**
Chu et al. [14]	**Prospective single-centre, double-blinded, parallel-group RCT**	**Strabismus**	**China (April to October 2020)**	**Intranasal dexmedetomidine vs no medication**	**10 min before separation from parents until anaesthesia induction**	**8% sevoflurane**	**2 µg kg**^**−1**^	**No medcation**	**70**	**70**
Elghamry and Elkeblawy [15]	**Prospective single-centre, double-blinded RCT**	**Strabismus**	**Egypt (November 2019 to April 2020)**	**Intravenous dexmedetomidine vs placebo**	**10 min before the end of the surgery**	**Sevoflurane**	**0.3 μg kg**^**−1**^	**0.9% saline**	**33**	**34**
Oriby and Elrashidy [16]	**Prospective single-centre, double-blinded, pparallel-groupRCT**	**Strabismus**	**Qatar (October 2018 to January 2020)**	**Intravenous dexmedetomidine vs propofol + remifentanil**	**Preoperatively**	**8% sevoflurane**	**1 mcg kg**^**−1**^	**4 mg kg**^**−1**^** h**^**−1**^** propofol + 0.03 mcg kg**^**−1**^** min**^**−1**^** remifentanil infusion**	**42**	**42**
Jangra et al. [17]	**Prospective single-centre, double-blinded, parallel-group RCT**	**Ophthalmic**	**India (April to October 2021)**	**Intranasal dexmedetomidine vs oral melatoni**	**45 min** **before the surgery**	**Sevoflurane** **5 to 8%**	**2 g kg**^**−1**^	**0.5 mg kg**^**−1**^	**60**	**60**
Li et al. [18]	**Prospective single-centre, double-blinded RCT**	**Strabismus**	**China (December 2018 to March 2019)**	**Intravenous dexmedetomidine vs placebo**	**Postoperatively**	**2 mg/kg propofol**	**0.3 μg. kg**^**−1**^	**0.9% saline**	**41**	**40**
Yao et al. [19]	**Prospective single-centre, double-blinded, parallel-group RCT**	**Strabismus**	**China (September 2013 to August 2014)**	**Intranasal dexmedetomidine vs placebo**	**45 min** **before the surgery**	**5% sevoflurane**	**2 mg kg**^**−1**^	**0.9% saline**	**51**	**52**

*D* dexmedetomidine group, *C* control group

**Table 2:** Baseline characteristics of 629 patients in the eight included studies

Baseline patient characteristics												
Ages, weight (median) and sex (no)									Procedure characteristics (min)			
Author and publication year	**Age**		**Male**		**Female**		**Weight (kg)**		**Surgery duration (min)**		**Anaesthesia duration (min)**	
	**D**	**C**	**D**	**C**	**D**	**C**	**D**	**C**	**D**	**C**	**D**	**C**
Ramlan and Mahri [12]	**3.00 (1.00–9.00)**	**5.00 (1.00–10.00)**	**16**	**15**	**16**	**17**	**13.00 (6.00–32.00)**	**18.00 (8.00–35.00)**	**N/A**		**60.17 ± 17.88**	**58.33 ± 19.31**
Sen et al. [13]	**3.77 (1–6)**	**3.42 (1–6)**	**27**	**23**	**13**	**17**	**14.5 (6–25)**	**13.7 (5–30)**	**19.5 ± 3.89**	**19 ± ** **4.11**	**29.88 ± 4.36**	**29.18 ± 4.21**
Chu and Wang, [14]	**4.73**	**4.7**	**31**	**36**	**39**	**36**	**N/A**		**N/A**		**N/A**	
Elghamry and Elkeblawy [15]	**4.65 ± 1.43**	**5.06 ± 1.37**	**11**	**14**	**23**	**19**	**17.1 ± 2.93**	**18.1 ± 2.88**	**49.6 ± 11.03**	**51.8 ± 9.64**	**59.8 ± 10.78**	**62.5 ± 9.63**
Oriby et al. [16]	**6.6 ± 2.5**	**5.8 ± 2.3**	**25**	**20**	**17**	**22**	**31 ± 12**	**29 ± 11**	**N/A**		**N/A**	
Jangra et al. [17]	**5.4 ± 2.0**	**5.5 ± 2.2**	**38**	**37**	**22**	**23**	**18.6 ± 5.7**	**17.5 ± 5.0**	**46.5 ± 2.3**	**47.1 ± 8.3**	**60.4 ± 3.5**	**60.3 ± 6.3**
Li et al. [18]	**8.25 ± 1.06**	**8.24 ± 1.32**	**20**	**24**	**20**	**17**	**N/A**		**22.70 ± 6.61**	**23.43 ± 7.86**	**32.53 ± 8.21**	**31.70 ± 7.58**
Yao et al. [19]	**4.9 ± 0.9**	**4.5 ± 1.0**	**30**	**37**	**22**	**14**	**19.3 ± 3.1**	**19.3 ± 4.2**	**44.0 ± 2.7**	**44.1 ± 3.3**	**84.3 ± 4.4**	**85.6 ± 4.5**

#### Bias risk assessment

RoB 1 was used to assess the methodological quality and bias risk of the eight included studies. Overall, the included studies had a low-bias risk in random sequence generation and allocation concealment since the randomisation procedure was clearly explained. The participants were randomised to the intervention and control groups using computer-generated randomisation in seven studies [13–19]. In one study, randomisation was performed by research team members not involved in data collection [12]. Therefore, selection bias risk was low in all the studies. Moreover, all studies clearly stated that anaesthesiologists, data collectors, patients and their families were blinded to the group assignment throughout the study. Therefore, the risk of performance and detection biases were low in all the studies. Finally, only one included study had selective reporting since they stated that PONV was more common in the control group than in the dexmedetomidine group [13]. However, they did not mention how many patients experienced PONV in both groups. Therefore, we rate this study as having a high-bias risk in selective reporting. We contacted the corresponding author, but they did not respond.

## Meta-analysis results

### ED incidence

**Fig. 4:**
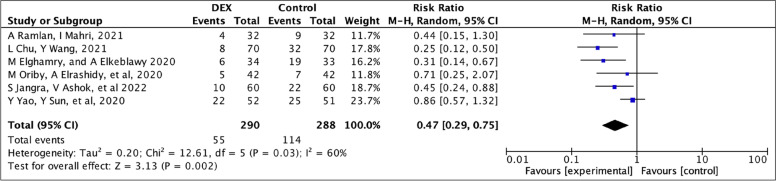
A forest plot of ED incidence. Weights are from a random effects analysis. Key: CI, confidence interval

Overall, six included studies reported the ED incidence [12, 14–17, 19] on the PAED scale, providing 578 patients for the meta-analysis of ED incidence. The ED rate was 39.5% in the control group and 18.96% in the dexmedetomidine group (RR=0.47; 95% CI 0.29–0.75). The overall effect size showed a significant difference between the dexmedetomidine and control groups (*z*=3.13, *p*=0.002). The *I*^2^ indicated moderate heterogeneity (*I*^2^=60%) (Fig. 4).

### Rescue analgesia

**Fig. 5:**
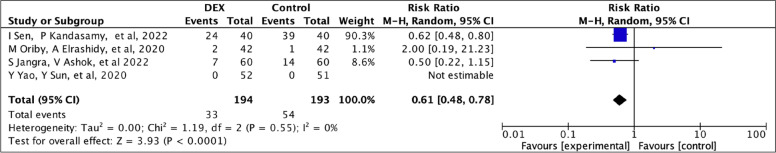
A forest plot for rescue analgesia use. Weights are from a random effects analysis. Key: CI, confidence interval

Four studies reported the use of rescue analgesia [13, 16, 17, 19], providing 387 patients for use in the meta-analysis. The results show a significant difference between groups in the use of rescue analgesia, with a *Z*-score based on the overall effect of (*p*=0.0001; RR=0.61; 95% CI 0.48–0.78). The percentage of patients who required analgesia was 17% in the dexmedetomidine group and 27.97% in the control group. The *I*^2^ indicated no heterogeneity (*I*^2^=0%) (Fig. 5).

### PONV

**Fig. 6:**
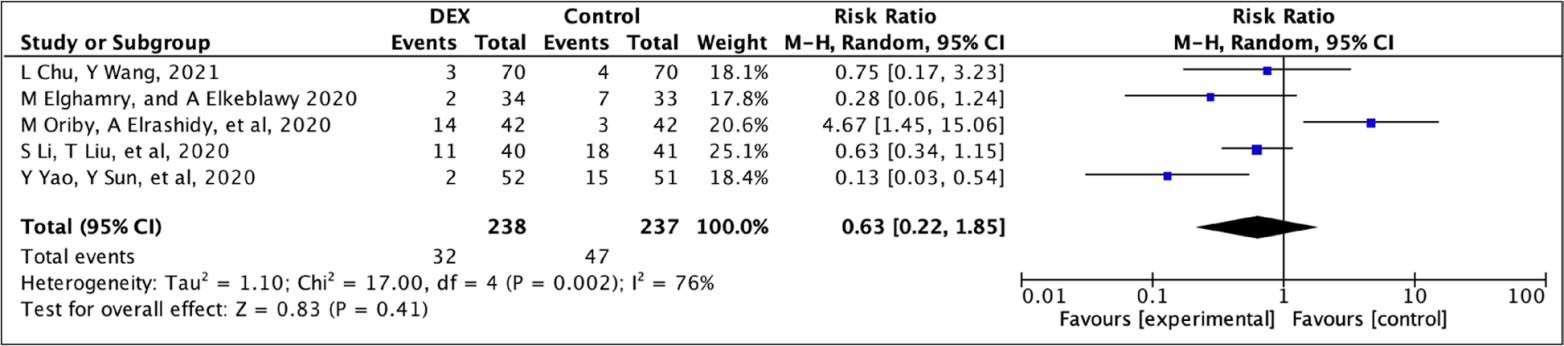
A forest plot of PONV incidence. Weights are from a random effects analysis. Key: CI, confidence interval

Five studies comprising 475 patients were included in the meta-analysis of PONV incidence [14–16, 18, 19]. The percentage of patients who experience PONV was 13.44% in the dexmedetomidine group and 19.8% in the control group (RR=0.63; 95% CI 0.22–1.85). The effect size showed no significant difference between groups (*p*=0.41). The *I*^2^ indicated high heterogeneity (*I*^2^=76%) (Fig. 6).

## Discussion

This systematic review and meta-analysis showed that dexmedetomidine significantly reduced ED incidence compared to placebo or other medications. Similarly, it reduced the use of rescue analgesia. However, dexmedetomidine did not help prevent PONV since no difference was found between groups.

A recent systematic review and meta-analysis done by Chiang et al. Included RCTs published before April 2020 to examine the efficacy of dexmedetomidine in preventing ED, PONV and postoperative pain in paediatric patients undergoing strabismus surgery [20]. Their results showed a significantly reduced in ED incidence (RR=0.39; 95% CI 0.25–0.62). Moreover, there was a significant reduction in analgesia use (RR=0.38; 95% CI 0.25–0.57). They also found that dexmedetomidine helped to prevent PONV (RR=0.33; 95% CI 0.21–0.54). Another recent systematic review and meta-analysis by Song et al. showed that intravenous dexmedetomidine helped to decrease ED incidence in paediatric patients undergoing strabismus surgery (WMD=3.05; 95% CI −3.82–2.27; *p*=0.017). Furthermore, dexmedetomidine reduced the incidence of postoperative vomiting (RR=0.28; 95% CI 0.13–0.61, *p*=0.001) [21]. The findings of both studies are consistent with our results except for PONV incidence, where our meta-analysis showed no difference between groups.

### ED incidence

Compared to general surgery, ophthalmic surgery is less traumatic, but ED incidence is nonetheless significant [17]. Patients undergoing ophthalmologic surgery had an ED risk rate of 28% compared to orthopaedic, urologic or other general surgery [12]. Using an eye patch to cover the operated eye increases anxiety and ED. Visual disturbances, fear of darkness and a lack of visual stimulation postoperatively can also lead to ED [17]. Moreover, anaesthetic choices, duration, surgery type and pain can contribute to ED occurrence [14, 15, 17]. As mentioned earlier, sevoflurane and desflurane are leading causes of ED, and both are commonly used in paediatric anaesthesia [4, 20]. A meta-analysis by Amorim et al. including RCTs that included paediatric patients undergoing elective procedures under general anaesthesia with sevoflurane found that dexmedetomidine helped to reduce the incidence of sevoflurane-induced ED compared to placebo [22]. However, in this review, seven included studies used sevoflurane for anaesthesia induction [12–17, 19], and one used propofol [18]. Therefore, we could not perform a subgroup analysis.

Five studies administered dexmedetomidine intranasally [12–14, 17, 19]. It is recommended to administer dexmedetomidine intranasally as a preoperative anxiolytic because it is noninvasive, facilitates parental separation, helps reduce the anxiety of paediatric patients in the operating room and ensures a smooth induction of inhalation anaesthesia [19]. Since the nasal mucosa is highly vascular, it provides a large surface area for drug absorption. Furthermore, nasally absorbed dexmedetomidine escapes first-pass hepatic metabolism, resulting in approximately 40% greater systemic bioavailability than the oral route [17, 23]. The bioavailability of intranasal dexmedetomidine using an atomiser was 83.8% in paediatric patients [22]. Furthermore, intravenous administration was marginally associated with bradycardia and hypotension due to its rapid effect than the intranasal route, which has a slower and more gradual onset [19]. Therefore, the intranasal route may be preferred over other routes [23].

### Rescue analgesia use in PACU

Postoperative pain is considered a significant contributing factor to ED, which is also associated with anaesthetic agents such as sevoflurane since many patients experience emergence agitation during recovery, mimicking ED [24].

Dexmedetomidine helped to reduce pain and prevent agitation and ED as it has both sedative and analgesic effects. The sedative effect of dexmedetomidine occurs through its interaction with postsynaptic α2-adrenergic receptors in the locus coeruleus, decreasing noradrenaline release and enhancing inhibitory neuron action, notably the gamma-aminobutyric acid system. The analgesic effect results from the effect of α2-adrenergic receptors on the dorsal horn and supra-spinal cord, reducing substance P release [22].

Previous studies have shown that most parents prefer a calm and sedated child in the immediate postoperative period. This preference is particularly important after ophthalmic surgery since crying or straining might increase intra-ocular pressure in the recently operated eye, potentially leading to adverse effects [17].

### PONV

In paediatrics, nausea and vomiting are major causes of postoperative discomfort [16]. PONV tends to be more common in general anaesthesia than in spinal anaesthesia [25]. Moreover, PONV in paediatric anaesthesia is associated with other risk factors, including surgery lasting >30 min, age ≥3 years, previous PONV, positive family history and strabismus surgery [18]. In addition, it can lead to electrolyte imbalance and extend the patient’s stay in the recovery room [25]. Several studies have recently focused on the effect of dexmedetomidine on PONV, finding that it has an antiemetic effect and may reduce PONV incidence [25, 26]. However, the optimal dose to achieve antiemetic effects remains unknown [18].

In our PONV meta-analysis, three included studies postoperatively administered ondansetron intravenously to all the patients in both groups [12, 15, 17]. In addition, one study administered ondansetron only in emesis cases [13], and another administered ondansetron to patients who experienced >2 emetic episodes or requested an antiemetic [18]. Our meta-analysis shows that dexmedetomidine did not reduce PONV incidence. However, variation in ondansetron administration time might affect the meta-analysis results, which might also explain the high heterogeneity indicated by the *I*^2^ test.

### Strengths and limitations

The strengths of this systematic review include the comprehensive search across databases for up to date evidence. Furthermore, we included only RCTs. All were prospective, double-blinded, parallel-group RCTs [12–19]. However, this review also had several limitations. The included studies administered dexmedetomidine as premedication before induction to provide sufficient time for the drugs to be absorbed. However, the premedication time was not always controlled, which may impact the drug effects [12]. Moreover, there was no long-term follow-up of patients diagnosed with ED [15].

## Conclusions

This systematic review and meta-analysis showed that the use of dexmedetomidine helped to reduce ED incidence in paediatric patients after ophthalmic surgery. Similarly, it reduced the need for rescue analgesia compared to placebo or other medications. However, there was no significant difference in PONV incidence between groups.

## Data Availability

Not applicable.
